# Contribution of the different *Neisseria gonorrhoeae* lipooligosaccharide structural variants to functional responses elicited by GMMA outer membrane vesicles

**DOI:** 10.1038/s41541-025-01271-1

**Published:** 2025-10-30

**Authors:** R. Cuffaro, G. Buffi, G. Romagnoli, M. Giuliani, D. Proietti, M. Zambelli, M. Spinsanti, A. G. O. Manetti, M. Fabbrini, S. Savino, G. Giordano, I. Delany, E. Bartolini, S. Ram, M. R. Romano, I. Margarit, F. Carboni

**Affiliations:** 1 GSK, Siena, Italy; 2 Division of Infectious Diseases and Immunology, University of Massachusetts Medical School, Worcester, MA USA

**Keywords:** Vaccines, Bacterial infection

## Abstract

Despite decades of research, an effective vaccine against *Neisseria gonorrhoeae* remains elusive due to the pathogen's antigenic variability and immune evasion capabilities. Retrospective studies of OMV-based meningococcal vaccines have shown a partial effectiveness against gonorrhea, reigniting hopes for a feasible vaccine. Our study focused on the role of gonococcal lipooligosaccharides (LOS), the most abundant antigens on the surface, in stimulating functional immune responses. By employing detoxified OMV from *Neisseria gonorrhoeae* isogenic strains expressing different LOS glycoforms, we observed that antibodies targeting LOS with long α-chain oligosaccharides correlated with bactericidal activity against a wide range of gonococcal isolates, whereas antibodies that also recognized the β-chain and consequently the 2C7 epitope could achieve a broader bacterial adhesion-inhibiting effect. The results obtained underscore the potential of targeting defined LOS structures to elicit cross-strain protective immunity. Insights from our findings may guide the design of vaccine strategies to combat the threat posed by antimicrobial-resistant gonorrhea.

## Introduction

Over the past decade, gonorrhea infections have become a serious global health concern. The causative agent *Neisseria gonorrhoeae* or gonococcus (GC) has been listed by the World Health Organization (WHO) as a high-priority pathogen for which new therapeutic and prophylactic tools are urgently needed^1^.

**Fig. 1:**
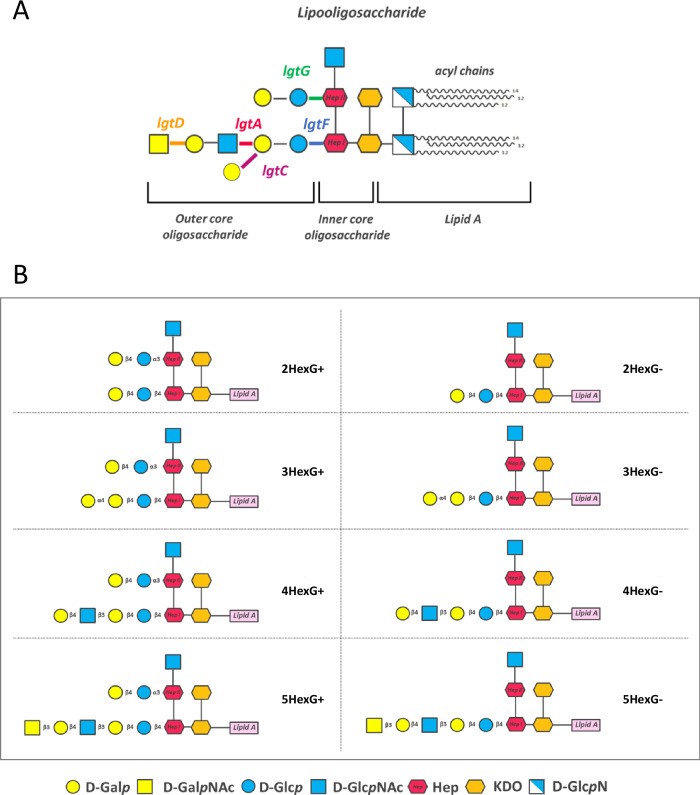
Schematic representation of gonococcal LOS structures and relevant *lgt* genes involved in their biosynthesis. **A** The figure illustrates all potential sugars and the *lgt* phase-variable genes (*lgtA*, *lgtC*, *lgtD*, and *lgtG*) that are responsible for the synthesis and expression of the Lgt enzymes and, therefore, of the diverse LOS structures. The *lgtF* gene, which is constitutively expressed and responsible for starting the synthesis of the α-chain, is also highlighted. **B** Schematic representation and nomenclature of the eight different LOS structures expressed by eight *Neisseria gonorrhoeae* MS11 *lgt* mutant strains^20^, whose LOS glycan structure varied based on the expression of the *lgt* phase-variable genes that were either fixed ‘on’ or ‘off’ in different combinations. Each mutant is designated by a number that indicates the length of its Heptose-I chain (2Hex, 3Hex, 4Hex, or 5Hex, corresponding to 2, 3, 4, or 5 sugars in the α-chain) and by G+ or G− labels that denote the presence or absence of the β-chain.

Out of the 376 million cases of sexually transmitted infections (STIs) globally reported in 2016, ~87 million were diagnosed with gonorrhea^2,3^. The escalating rise in the incidence of infections and the worldwide spread of multidrug-resistant strains emphasize the critical importance of developing new antibiotics and vaccines. Natural immune responses from infection fail to prevent reinfection, and vaccines investigated to date in humans have not provided acceptable efficacy^4^. A seminal retrospective observational study reported a 31% reduction in gonococcal infections after a national immunization program in New Zealand using the MenB outer membrane vesicle (OMV) vaccine MeNZB^5^. Subsequent similar studies with vaccines containing MenB OMV, including 4CMenB (Bexsero)^6^, have confirmed a reduction of gonorrhea by 31–40%, and efforts have been directed towards the development of gonococcal OMV-based vaccines^7,8,9^. A vaccine candidate, called NgG, based on gonococcal generalized modules of membrane antigens (GMMA), was recently shown to elicit antibodies in mice mediating bacterial killing of a wide range of strains^10^. GMMA are outer membrane vesicles released from microorganisms genetically modified to enhance certain desired characteristics, such as reduced reactogenicity of the lipid A moiety of surface lipopolysaccharides (LPS) or lipooligosaccharides (LOS), the removal of undesired antigens and/or the increased release of vesicles^11^. The study highlighted that LOS was essential for the induction of cross-functional antibodies by the NgG vaccine in mice, which were not observed when using an analogous GMMA from a strain lacking the *lgtF* gene, encoding the enzyme responsible for attaching the first galactose residue to Heptose I (Fig. 1A) and therefore devoid of the major LOS glycan epitopes^10^.

Among the gonococcal surface antigens, the oligosaccharide component of LOS has been widely explored as a potential vaccine target, primarily due to its dense presentation on the bacterial surface and high accessibility as a target of adaptive immunity^12^. LOS mediates several mechanisms of gonococcal infection, such as serum resistance, cell adhesion and invasion, and escape from the host immune system^13,14^. Moreover, antibody responses to LOS have been shown to mediate complement activation, bactericidal and opsonic activities^15^. In particular, a promising LOS epitope known as 2C7 has been reported to be widely present across strains, to elicit cross-reactive bactericidal antibodies in mice and to attenuate gonococcal vaginal colonization in a mouse model of infection, supporting the potential use of LOS as a vaccine candidate^16,17^.

The synthesis of LOS glycan extensions is modulated by glycosyltransferases encoded by four phase variable genes (*lgtA*, *lgtC*, *lgtD*, and *lgtG*), the expression of which can be reversibly switched on and off. The phase variable mechanism involves slipped-strand mispairing during bacterial replication at homopolymeric tracts within the coding regions of these *lgt* genes, enabling strain subpopulations to express multiple antigenically different structures^18^, and thus aiding the bacteria to adapt to different host environmental conditions^19^. In particular, the *lgtA*, *lgtC*, *lgtD* genes modulate the sugar composition of the chain extending from the Heptose I (HepI), known as alpha (α) chain, while the *lgtG* gene product can modify the glycan extensions from Heptose II (HepII) or beta (β) chain, resulting in eight different structures that vary according to the length of their α-chain and presence or absence of the β-chain (Fig. 1). These LOS structures can be expressed individually or co-expressed in diverse combinations by each gonococcal strain^20^.

Moreover, *N. gonorrhoeae* acquires host-derived CMP-Neu5Ac to sialylate terminal LOS galactose moieties and is also capable of introducing phosphoethanolamine (PEA) modifications in the LOS lipid A. Both modifications impair complement activation, enabling the bacteria to evade host immunity^13,21–23^.

The present study was aimed at assessing the impact of the different LOS glycoforms on eliciting functional immune responses. In particular, we investigated the functional activity of antibodies induced by GMMA derived from strains expressing different LOS variants, in terms of bacterial killing and inhibition of host cell adhesion.

## Results

### LOS heterogeneity in a panel of gonococcal isolates

We recently demonstrated that a vaccine based on *N. gonorrhoeae* FA1090 GMMA (NgG)-induced antibody responses in mice mediating bactericidal activity against a heterologous panel of strains previously selected to be representative of the gonococcal surface protein heterogeneity^10^. In that study, GMMA derived from an isogenic *lgtF* mutant devoid of the major LOS epitopes, as the *lgtF* gene is responsible for the initial addition of monosaccharides to the α-chain from the core heptose HepI, induced significantly less cross-functional responses, pointing towards an important role of anti-LOS antibodies.

**Fig. 2:**
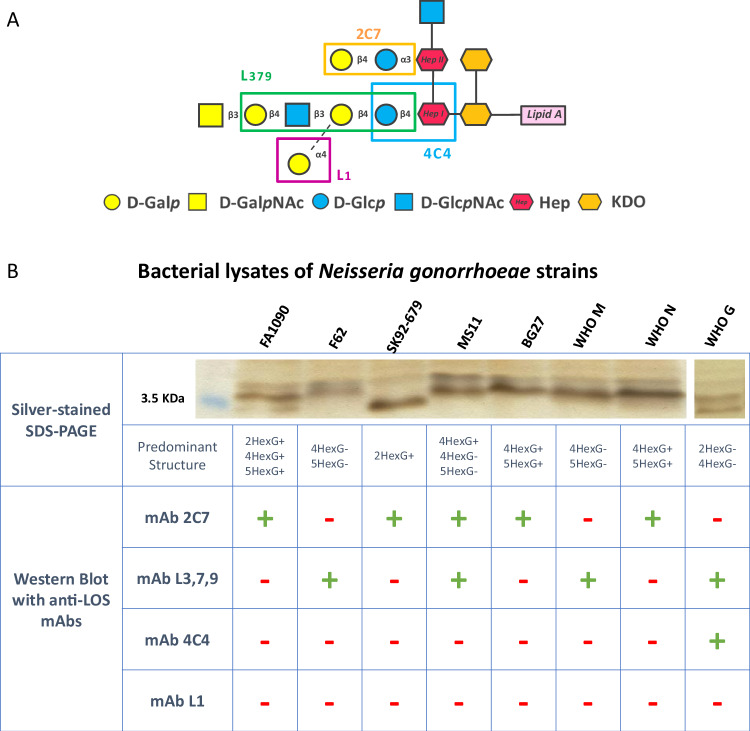
Characterization of the LOS of the *Neisseria gonorrhoeae* strains used in this study. **A** LOS epitopes recognized by L1, 4C4, L3,7,9 and 2C7 mAbs used for the LOS characterization of the different *Neisseria gonorrhoeae* strains^24–26^. **B** Prevalent LOS structures expressed by a panel of gonococcal strains determined by immunochemical characterization. LOS structures were attributed according to the pattern and the MW height of the bands in the silver-stained SDS–PAGE analysis combined with the recognition of selective anti-LOS antibodies detected by Western Blot. Images from two distinct gel cuts have been merged; white space indicates boundaries between each gel. Uncropped and unprocessed scans of the gels are provided in Supplementary Fig. 5.

We first assessed the diversity of oligosaccharide epitopes expressed by eight strains of a gonococcal strain panel used in this study. Heat-inactivated whole bacterial cell lysates were analyzed by silver stained SDS–PAGE and probed by Western Blot using four monoclonal antibodies recognizing different LOS epitopes, i.e. mAb L1^24^, 4C4^25^, L3,7,9^24^ and 2C7^26^ (as depicted in Fig. 2A). The observed pattern of low molecular weight bands combined with specific mAb recognition enabled the identification of the predominant LOS structures expressed by each strain (Fig. 2B).

Most of the isolates expressed multiple LOS structures presenting as multiple bands in SDS–PAGE that were recognized by more than one mAb, which allowed for a detailed characterization of their LOS isoform combinations. In particular, FA1090 displayed a pattern indicative of LOS structures containing a β-chain (G+) recognized by mAb 2C7 and differentiated by the length of the α-chain, from 2 to 4 and 5 sugars (2HexG+, 4HexG+, and/or 5HexG+). Four additional strains expressed G+ LOS structures identified by mAb 2C7, which has been reported to be widely expressed across minimally passaged gonococcal isolates^22,26^. Four of the strains expressed the 4HexG- lacto-*N*-neotetraose (LNnT) structure, recognized by the meningococcal mAb L3,7,9, also very frequently expressed by gonococci^22,26^. The WHO-G strain was the only one additionally expressing the shorter 2HexG- structure characterized by a lactose (Galβ1-4Glc) extending from the Heptose-I. None of the strains expressed 3Hex-like structures (Galα1-4Galβ1-4Glc in the α-chain) recognized by mAb L1, which have been reported as poorly represented in circulating gonococcal isolates^22,27,28^.

Overall, the obtained results confirmed a significant variability in LOS structures among the selected *N. gonorrhoeae* strains and suggested that the panel could be considered representative of the broad LOS heterogeneity.

Two bacterial strains with distinctly defined LOS composition were selected to initially investigate the impact of the β-chain and of long α-chain structures on the elicitation of functional antibodies by their derived GMMA. The first strain, F62, lacks the β-chain and exclusively expresses a long α-chain with 4HexG− or 5HexG− structures. The second strain, SK92-679, has a complete two-sugar β-chain branching from Heptose-II but lacks long α-chain structures.

### Gonococcal GMMA with long LOS α-chain structures elicits high bactericidal titers against multiple GC strains

To ascertain which of the LOS structures predominantly contribute to the induction of broadly functional antibodies, we analyzed immune responses in mice vaccinated with GMMA obtained from the two selected isolates expressing distinct LOS structures, F62 and SK92-679 (as described above). Following genetic detoxification by removal of the *lpxL1* gene, which resulted in the expression of a penta-acylated lipid A, GMMA were isolated, purified and immunochemically characterized. The analysis confirmed the exclusive presence of LOS with long α-chain structures in the absence of the β-chain in GMMA from the F62 Δ*lpxL1* strain, and of a single predominant 2HexG+ LOS structure with two sugars in the α-chain and two in the β-chain in vesicles obtained from SK92-679 Δ*lpxL1* (data not shown).

**Fig. 3:**
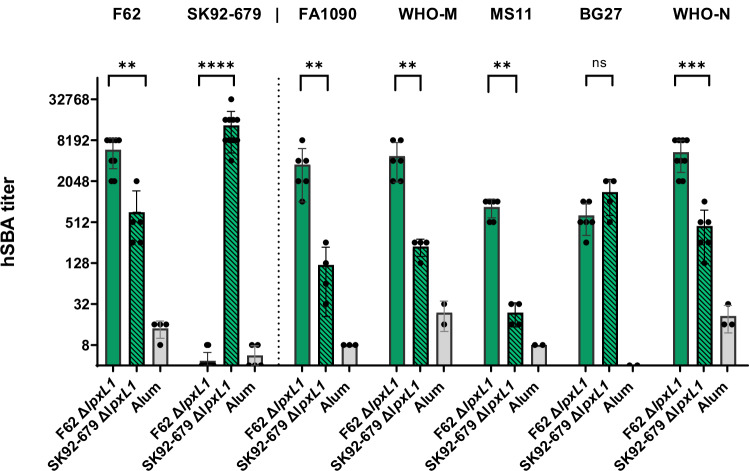
hSBA titers against the gonococcal strains indicated on top, obtained using sera from CD1 mice immunized with Alum-adjuvanted GMMA F62 Δ*lpxL1*, GMMA SK92-679 Δ*lpxL1* or Alum alone. Each bar represents the mean titer obtained with serum pools from three (GMMA F62 Δ*lpxL1*) or two (GMMA SK92-679 Δ*lpxL1*) independent groups of 10 mice immunized with different batches of GMMA; the standard deviation of the mean is indicated. Each dot represents the titer derived from three experimental replicates for each serum pool. The Mann–Whitney test was used to compare GMMA F62 Δ*lpxL1* and GMMA SK92-672 Δ*lpxL1* immunization groups (**P < 0.01; ***P < 0.001; ****P < 0.0001). A pool of sera from mice immunized with Alum was used as a negative control.

These GMMA were subsequently tested in an in vivo study, where female outbred mice were immunized twice with 10 µg of GMMA (based on the protein content) adsorbed on alum hydroxide. Adjuvant alone was tested as a negative control. Pooled sera were collected two weeks after the second dose and tested in a human Serum Bactericidal Assay (hSBA) against the corresponding homologous strains plus six additional gonococcal strains from the above panel (Fig. 3).

Each GMMA-induced bactericidal antibodies towards the homologous strain and at least five out of six heterologous strains compared to Alum. Furthermore, sera raised against F62 Δ*lpxL1* GMMA showed at least 1-log higher bactericidal titers on five out of the seven strains tested compared to the SK92-679 Δ*lpxL1* GMMA antisera (*P* < 0.01). This observation suggested a potential role of distinct LOS structures in eliciting highly functional antibodies, and the potential importance of the long α-chain or presence/absence of the β-chain in the induction of broad and high bactericidal activity, although a contribution of protein antigens present in the GMMA from different background strains could not be excluded from these experiments.

Building on the results obtained, further experiments were conducted using a panel of isogenic LOS mutants derived from a single strain. This approach aimed to assess the impact of individual LOS structures within a consistent protein profile background, thereby offering a clearer understanding of their role in eliciting broad bactericidal responses.

GMMA were prepared from the eight isogenic *lgt* mutant strains derived from GC MS11, each expressing a defined LOS structure^20^ (as depicted in Fig. 1). Each of the mutant strains was genetically engineered to remove the *lpxL1* gene for detoxification and immunochemically characterized to confirm its LOS pattern. The expression of LOS in the derived GMMA (Supplementary Fig. 1) matched the LOS present on the respective non-detoxified strain described by Chakraborti et al*.*^20^*,* indicating that neither the absence of one of the Lipid A acyl chains nor the multiple plate passages during transformation affected the overall LOS phenotype.

GMMA from each individual mutant was adsorbed to Alum hydroxide and tested by immunizing female outbred mice. Here, the dose of GMMA was normalized based on the LOS content to 1.5 nmol. Doses in terms of protein quantity are reported in Supplementary Information (Supplementary Table 1) and ranged from 3.9 to 9.6 μg.

**Fig. 4:**
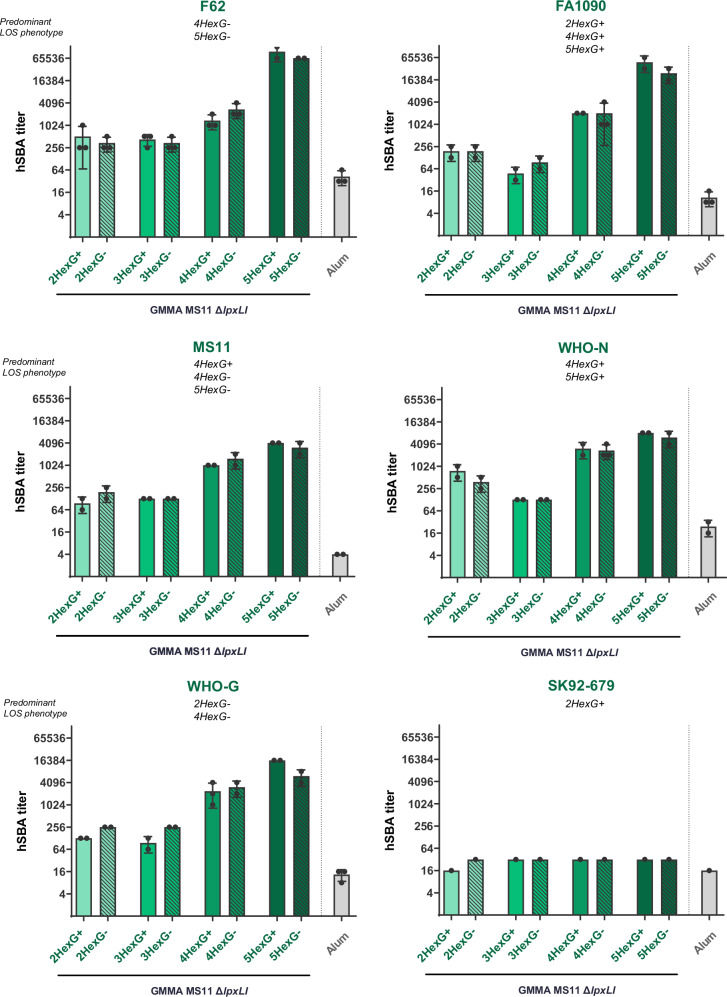
hSBA titers measured against the gonococcal strains indicated on top, using pooled sera from CD1 mice immunized twice with GMMA MS11 Δ*lpxL1* mutants. Each bar represents the mean titer derived from at least two experiments conducted with serum pools from groups of 10 mice immunized with GMMA from each of the mutant strains; the standard deviation of the mean is indicated. Each dot represents the titer derived from each independent experimental replicate for a specific serum pool. A pool of sera from mice immunized with Alum was used as a negative control.

Sera were collected two weeks after the second dose and analyzed in pools by hSBA. Bactericidal activity was tested using a sub-panel of GC strains, including the homologous MS11 strain and five heterologous strains covering various LOS structures. The results reported in Fig. 4 showed that all the variants of the MS11 GMMA induced a bactericidal response against four out of five tested strains, which was consistently higher than the negative control, represented by Alum hydroxide alone. Interestingly, sera from mice immunized with GMMA containing long α-chain LOS structures (4HexG+/G−; 5HexG+/G−) exhibited significantly higher bactericidal activity than sera from mice immunized with those presenting shorter LOS structures (2HexG+/G−; 3HexG+/G−). This trend was observed in five out of the six *N. gonorrhoeae* strains tested, except for SK92-679, where none of the sera yielded bactericidal responses above negative control levels. Overall, these results suggested that the length of the α-chain was a crucial parameter, whereas expression of the β-chain did not appear to impact the elicitation of bactericidal antibodies.

### Cross-bactericidal activity of antibodies induced by NgG mainly depend on long LOS α-chain epitopes

We further investigated whether cross-functional activity elicited by GMMA derived from a strain containing multiple LOS structures like FA1090 could mainly be ascribed to antibodies directed against long α-chain epitopes. To address this, a competitive hSBA experiment was developed, where mouse serum from animals immunized with NgG^10^ was pre-mixed with GMMA derived from the eight MS11 *lgt* mutant strains. The serum was then tested against four gonococcal strains expressing diverse LOS structures (WHO-N, WHO-G, MS11, and F62). Three different dilutions of competitors, normalized for LOS concentration (nmol/mL), were tested to confirm the response specificity.

**Fig. 5:**
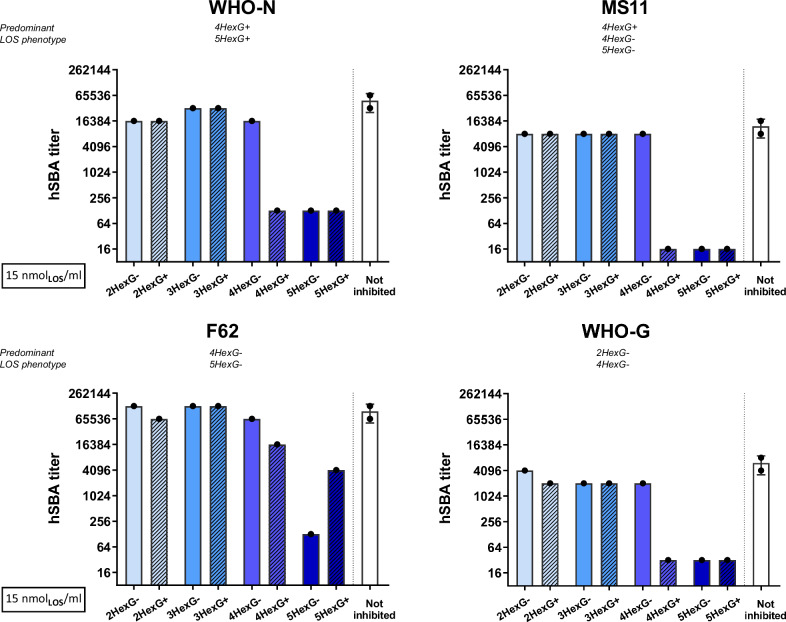
Competitive hSBA titers obtained using sera from mice immunized with GMMA FA1090 Δ*lpxL1* Δ*rmp* (NgG) after preincubation with a 15 nmol_LOS_/ml concentration of GMMA from MS11 mutant strains. Each bar/dot represents the titer obtained using a pool of sera from 10 mice immunized with GMMA FA1090 Δ*lpxL1* Δ*rmp* and competed with GMMA from the MS11 mutants reported on the *x*-axis. The white bar represents the uninhibited sample, tested in duplicate.

Bactericidal titers against the heterologous strains, measured in the presence of high concentration of LOS (present in GMMA) as a competitor, are presented in Fig. 5. As shown in Supplementary Fig. 2, a dose-dependent inhibition was obtained with 4HexG+, 5HexG−, and 5HexG+ competitor GMMA for all strains except F62, which was not completely inhibited by 4HexG+ and 5HexG+ when tested at the highest concentrations. Of note, F62 is intrinsically highly sensitive to serum and exhibited extremely high SBA titers and lower inhibition across all competitor GMMA concentrations compared to other tested strains. We hypothesize that higher inhibition would have been observed also for F62 if a higher concentration of competitor GMMA had been used.

In all cases, antibody functional activity was inhibited by GMMA with a long α-chain of 4 and/or 5 sugar residues, corresponding to structures 4HexG+, 5HexG− and 5HexG+, presumably by competitive binding. Interestingly, the 4HexG− did not outcompete the bactericidal activity against any of the strains. This contrasts with observations from direct immunization using MS11 isogenic strains and may be explained by the LOS composition of the NgG. The NgG predominantly presents 2HexG+, 4HexG+, and 5HexG+ structures, thereby likely stimulating antibodies that target LOS structures containing β-chain. As a result, it is probable that no/few antibodies against the 4HexG− structure are elicited, which could explain the absence of hSBA inhibition. To corroborate this hypothesis, we conducted Western Blot analyses using anti-FA1090 serum to test recognition of MS11 GMMA 4HexG+ and 4HexG− components. While the high molecular weight protein bands displayed comparable profiles between the two GMMA preparations, there was a strong difference in the intensity of the low molecular weight LOS signals, with the 4HexG+ GMMA showing a pronounced band and 4HexG− a faint band. The data confirmed that FA1090 GMMA elicited minimal responses against 4HexG− LOS structures (Supplementary Fig. 3). The situation might differ for the 5HexG− structure, able to inhibit the bactericidal activity, as it might interfere with antibodies targeting the terminal GalNAc residue in the α-chain, the epitope recognized by the anti-LOS mAb 1-1-M^29^, whose binding could potentially be unaffected by the presence or absence of the β-chain. We speculate that the lack of a β-chain in 5HexG− GMMA can make the epitope more accessible, reducing steric hindrance and possibly leading to better inhibition of this class of antibodies.

Conversely, structures with short α-chains containing only 2 or 3 sugars linked to Heptose-I, were unable to inhibit bactericidal activity. Therefore, the results further supported the hypothesis that antibodies against LOS structures with long α-chains were responsible for the cross-bactericidal activity elicited by gonococcal GMMA.

### Assessment of the effect of variable glycan chains on the inhibition of bacterial adhesion by gonococcal GMMA-elicited antibodies

**Fig. 6:**
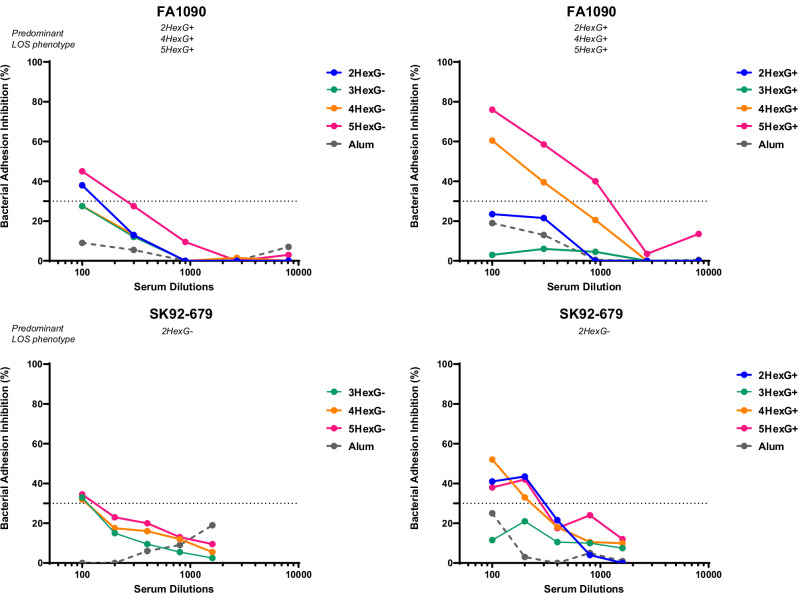
BAI measured on SV-HUC1 cells against FA1090 and SK92-679, obtained using pooled sera from CD1 mice immunized twice with the indicated GMMA MS11 mutants (G− mutants in the left panels and G+ mutants in the right panels). Each dot represents the median value obtained from at least four independent determinations. A pool of sera from mice immunized with Alum was used as a negative control.

The potential impact of antibodies targeting specific individual LOS structures was also evaluated for their capability to inhibit the adhesion to the human SV-HUC1 cell line representative of the male urogenital epithelium using a bacterial adhesion inhibition (BAI) assay on two strains, FA1090 and SK92-679, selected for their distinct LOS structures. Sera raised against a subset of the GMMA from MS11 *N. gonorrhoeae* strains with distinct LOS structures were tested for their ability to inhibit bacterial adhesion to cells (Fig. 6).

The role of anti-LOS antibodies was evident for both tested strains; distinct functional responses were observed for antibodies raised against different LOS structures, as measured by their ability to inhibit adhesion above the 30% threshold. For both target strains, the data indicated that antibodies raised against G− GMMAs exhibited an almost negligible functional response compared to antisera from G + GMMA-immunized mice. This was expected, given that G− strains were less relevant for FA1090 and SK92-679 strains, which predominantly express G+ structures. However, as shown in Fig. 6, we observed some differences among GMMA expressing different G + LOS variants, with a different trend for the two tested strains. In the case of FA1090, which predominantly expresses 2HexG+, 4HexG+ and 5HexG+ structures, GMMA displaying LOS with a long α-chain (4HexG+ and 5HexG+) elicited antibodies with functional activity, while antibodies elicited by LOS presenting only 2 or 3 sugars linked to Heptose-I (2HexG+ and 3HexG+) were unable to inhibit bacterial adhesion to epithelial cells. For SK92-679, which predominantly displays a 2HexG+ like structure (Fig. 2), antibodies elicited from 2HexG+ resulted in a comparable BAI titer to antibodies from GMMAs with longer α-chains (4HexG+ and 5HexG+), demonstrating that antibodies to 2HexG+ structures can inhibit bacterial adhesion, in contrast to their lack of function against this strain in SBA. This observation highlighted that the functional role of antibodies varies based on the specific LOS structures present in the tested strains.

It is noteworthy that antibodies elicited by GMMA with long α-chains could inhibit the binding of both strains, including SK92-679, composed almost entirely of short α-chains, which suggests that these GMMA-generated antibodies target shorter structures as well. To corroborate this hypothesis, we conducted Western Blot analyses using anti-MS11 4HexG+ serum to test recognition of MS11 GMMA mutant components. As shown in the Supplementary Fig. 4, a pronounced band corresponding to the 2HexG+ structure confirmed that these GMMA were able to elicit antibodies recognizing LOS with short α-chains. Given that the anti-MS11 4HexG+ antiserum poorly stained 2HexG− LOS, we conclude that the epitopes recognized by this antiserum also require part or all of the β-chain. In conclusion, antibodies targeting specific LOS structures vary in their ability to inhibit bacterial adhesion, and this largely depends on the LOS expressed by the target strain.

## Discussion

Despite decades of extensive research and the exploration of various potential candidates^9,30^, at present, there is no gonococcal vaccine available^4^. During the years, progress on gonorrhea vaccines has been challenging for several reasons, including the antigenically variable nature of this pathogen, its ability to evade host immune defenses and the unclear mechanism of protection against infection^31^. Immune responses triggered by primary infections do not provide protection against subsequent disease, antibody responses to some antigens such as Reduction modifiable protein (Rmp) may lead to inauspicious responses^32^, and historical candidates that progressed to clinical studies were unsuccessful^33,34^.

Recent clinical observations evidenced that cross reactivity between *N. meningitidis* and *N. gonorrhoeae* antigens may induce cross protection, as suggested by the moderate effectiveness of meningococcal OMV-based vaccine approaches on gonorrhea^35,36^. However, the mechanism beyond the meningococcal vaccine protective activity is still not fully understood. Among the main gonococcal membrane surface components evaluated as potential target antigens, much research has been carried out on LOS, primarily focusing on the impact of this antigen on the pathogenicity, host-adaptation and protection against immune responses^12^. The 4CMenB vaccine appears to stimulate the production of bactericidal antibodies that can target LOS epitopes found on both *N. meningitidis* and *N. gonorrhoeae* and that could potentially support the efficacy of meningococcal OMV vaccines in the protection against gonococcal infections^37^. Very recently, by isolating human monoclonal antibodies from vaccinees, some of these LOS epitopes have been identified^38,39^

Gonococcal LOS glycan expression is highly variable due to phase variation of four glycosyltransferase genes (*lgtA*, *lgtC*, *lgtD*, and *lgtG*) responsible for the synthesis of the oligosaccharide chains, leading to the expression of different structures across strains and even within a single bacterial strain^19^. In the present study, we investigated the functionality of antibodies raised to the diverse LOS structures using GMMA technology.

Given the various pathogenic mechanisms of GC infection, it is plausible that an effective vaccine would produce antibodies mediating complement-dependent bacterial killing and/or prevent bacterial adhesion. Indeed, despite the absence of a defined correlate, recent mouse studies suggest that complement-mediated killing might be a key protective mechanism against gonococcal infections^17,40,41^. Therefore, to compare the functional efficacy of antibodies induced by different LOS structures, a human serum bactericidal assay and a bacterial adhesion inhibition assay using urethral epithelial cells were used.

The role of each antigenically distinct LOS isoform in the functional immune response induced by a GMMA-based vaccine was initially investigated by examining two different strains with distinct LOS patterns (F62 and SK92-679) and subsequently by using a panel of isogenic strains expressing predominantly a single LOS structure that could not phase vary^20^. Results indicated that LOS α-chains composed of four or more monosaccharides (4Hex or 5Hex) elicited higher bactericidal titers against all tested strains, compared to those elicited by short LOS α-chains, except for SK92-679, which was not killed by any MS11 antisera. The obtained data are consistent with earlier findings showing that meningococcal antibodies purified against long LNnT LOS structures bound multiple epitopes within the LNnT framework and correlated with significant bactericidal activity^42,43^. The absence of bactericidal activity against SK92-679 was previously reported for antibodies elicited by an FA1090-derived GMMA vaccine (NgG), while immunization with the homologous SK92-679 GMMA demonstrated significant bactericidal activity against this strain^10^. These data suggest the presence of alternative bactericidal targets against SK92-679, potentially involving proteins that are not expressed by MS11 and FA1090 strains. Considering that SK92-679 is a PorB1a strain, whereas all other GMMA tested have been produced from PorB1b strains, we cannot exclude the possibility of PorB being the protective antigen. LOS sialylation is known to be very important in gonococcal susceptibility to complement-mediated bactericidal activity. While CMP-NANA was used to confer serum resistance to three of the strains used (F62, MS11 and WHO-M), the role of sialic acid has not been evaluated in detail in the presented experiments.

Moreover, we assessed whether antibodies generated against MS11 *lgt* mutant strains could prevent bacterial adhesion to SV-HUC1 urethral epithelial cells using two different bacterial strains, FA1090 and SK92-679. Our results indicate the significance of anti-LOS antibodies in impeding bacterial adhesion in both strains. For the FA1090 strain, antibodies were particularly effective when generated using GMMA enriched with LOS featuring a long α-chain and the β-chain. Conversely, the SK92-679 strain showed comparable antibody activity with GMMA expressing either a LOS with a long α-chain or the 2HexG+ short structure. Our findings for the FA1090 strain align with previous research suggesting that LOS structures containing the unsialylated LNnT epitope engage with the asialoglycoprotein receptor, thereby facilitating gonococcal adhesion to male urethral epithelial cells^44^. Interestingly, SK92-679 exhibited a distinct behavior, suggesting that antibodies against the 2C7 epitope (present in 2HexG+ structures) may also inhibit bacterial adhesion to male urethral cells. This suggests that the effectiveness of antibodies in inhibiting bacterial adhesion is not solely dependent on the GMMA variants used for immunization, but also on the LOS structure present on the surface of the target strain.

Previous studies have demonstrated a natural selection for long α-chain LOS immunotypes during urethral infection in men^45^. The presence of LNnT is crucial for LOS-mediated adherence to male urethral epithelial cells^44^, as it also plays a role in facilitating LOS-mediated invasion into human cervical epidermoid cell lines^14^. Indeed, the LNnT epitope has been reported to be involved in immune evasion strategies, increased virulence and pathogenicity^14,46,47^. Moreover, immunochemical characterization of LOS in 75 minimally passaged clinical isolates from Nanjing, China, revealed that all strains expressed not only the 2C7 epitope but also LNnT, as demonstrated by their reactivity with monoclonal antibody 3F11^22^. Therefore, while more detailed phenotypic analyses of LOS structures in circulating strains are crucial for evaluating vaccine coverage, existing data indicate that long α-chain structures are prevalent, making them promising candidates for vaccine development.

Targeting LOS long α-chain structures containing the LNnT epitope has raised potential concerns due to its resemblance to human paraglobosides. However, existing data indicate that antibody interactions with LNnT are influenced by the LOS lipid moiety, which differs from human ceramide lipids, thereby preventing autoimmune binding^48^. Additionally, no safety signals have been reported after extensive use of Neisserial OMV-based vaccines, supporting the consideration that LNnT-containing structures could be a viable target for vaccine development.

In conclusion, the insights gained from this study may further support the importance of considering LOS as an important antigen for induction of possible protective antibodies against *N. gonorrhoeae* and provide a basis to support the elucidation of the role of different LOS isoforms to unravel the GMMA-based vaccine mechanism of action.

## Methods

### *Neisseria gonorrhoeae* strains for GMMA production and functional assays

*Neisseria gonorrhoeae* strains were routinely cultured at 37 °C in an atmosphere of 5% CO_2_ on Gonococcus (GC) agar medium plates enriched with 1% v/v of Isovitalex. For liquid cultures, bacteria from a fresh overnight plate culture (16 ± 2 h) on the GC agar medium plates 1% Isovitalex were diluted to reach a starting optical density at 590 nm (OD_590_) equal to 0.3–0.4 in liquid GC—1% v/v Isovitalex, supplemented if needed, and incubated at 37 °C at 160 rpm. Cytidine-5’-*O*-monophospho-*N*-acetylneuraminic acid [CMP-NANA] was not added to the broth medium used for these liquid cultures. When required, to induce pilus expression and enable transformation, GC strains were cultured on GC agar plates supplemented with 0.25 mM (IPTG).

### Neisseria gonorrhoeae MS11 LOS lgt mutant strains

*Neisseria gonorrhoeae* MS11 LOS *lgt* mutant strains used in this work consist of eight isogenic *N. gonorrhoeae* mutant strains created in the background of *N. gonorrhoeae* MS11 4/3/1, a variant of MS11 VD300 with an isopropyl-D-thiogalactopyranoside (IPTG)-inducible *pilE* that controls pilus expression^20^. As described in Chakraborti et al*.*^20^, in this series of mutants, the glycan extensions from LOS core heptoses (Heptose-I and Heptose-II) have been modulated genetically, setting on or off (or deleting) the phase variable expression of the four LOS glycosyltransferase loci: *lgtA, lgtC* and *lgtD* genes, responsible for the variation in the α-chain extensions, and the *lgtG* gene, which controls the expression of the β-chain (a lactose on the Heptose-II). The resulting LOS structure of the mutant strains is reported in Fig. 2.

### Generation of *Neisseria gonorrhoeae* knockout strains

To reduce the endotoxin activity in the strains used, the *lpxL1* gene was deleted as in Spinsanti et al*.*^10^ for SK92-679 and F62, while the Δ*lpxL1* cmR plasmid containing upstream and downstream regions of the gene flanking a cloramphenicol resistance cassette was used in the collection of MS11 mutant strains. The plasmid was synthesized by GeneArt (Thermo Fisher Scientific) and transformed into DH5α *E. coli* competent cells (Thermo Fisher Scientific). Plasmid DNA was purified using HP Plasmid Mini kit II (Omega Biotek) according to the manufacturer’s instructions, and it was used as a template for a PCR amplification using primers LpxL1 UP Fwd and LpxL1 DO Rev. All primers used in this study are reported in Supplementary Table 2. The PCR product was purified using the Wizard SV Gel and PCR clean-up system (Promega) following the manufacturer’s protocol and then used for the transformation of the eight MS11 mutant strains. Transformations were carried out by spotting a mixture of bacterial resuspension in phosphate-buffered saline (PBS) and DNA onto a GC agar + 1% isovitalex plate and by incubating it for 5–6 h. Transformants were selected onto GC agar + 1% isovitalex plate with either kanamycin (70 µg/ml for F62 and 150 µg/ml for SK92-679) or cloramphenicol 10 μg/ml. To check the correct event of double recombination, transformants were tested by PCR using the Accuprime Taq Polymerase (Thermo Fisher Scientific) and with external primers LpxL1 est Fwd and LpxL1 est Rev. Positive clones were streaked repeatedly onto selective agar plates and glycerol stocks and DNA lysates were collected at each passage to test for the presence of remaining wild-type (WT) population. PCR screenings were performed using Accuprime Taq Polymerase and with the primer LpxL1 est FW in combination with the primer NGO_lpxL1_wtcheck-Rev, specific for the WT DNA. Absence of a PCR product would indicate the proper removal of all WT cells from the mutant clone.

### Bacterial fermentation

For each batch, bacteria from a fresh overnight plate culture (16 ± 2 h) on the GC agar medium plates 1% Isovitalex were diluted to reach a starting optical density at 590 nm (OD_590_) equal to 0.3–0.4 in liquid GC—1% v/v Isovitalex and incubated at 37 °C at 180 rpm until an OD_600nm_ equal to 1.5 ± 0.5 was reached. GMMA preparations were obtained from bacteria grown in absence of CMP-NANA.

### GMMA isolation and purification

The collected growth was centrifuged at 8000×*g* for 15 min at 4 °C. and the cell-free supernatant was then carefully recovered, filtering the solution through a 0.2 μm Sartobran P H9 filter to ensure bacteria removal. Then, MgCl_2_⋅6H_2_O was added to a final molarity of 10 mM and 50 U/L of benzonase was added for DNA removal and incubated at 4–8 °C while stirring overnight. A tangential flow filtration step using 200 cm2 300 kDa cut-off PESU membrane (Sartocon Slice 200 Sartorius stedim polyethersulfone 300 kDa) was used for retention of the GMMA and buffer exchange in PBS, followed by an ultracentrifugation step (150,000×*g* for 2 h) to pellet the GMMA, which were subsequently resuspended in PBS and sterile filtered using a 0.2 µm filter, to obtain final samples.

### GMMA characterization

Total protein quantification and protein concentration were performed with Pierce Modified Lowry Protein Assay from Thermo Scientific. The purity of the GMMA preparations was assessed by sodium-dodecyl-sulfate polyacrylamide gel electrophoresis (SDS–PAGE) and performing size exclusion chromatography module (SE-HPLC) to determine the purity using fluorescence to monitor soluble protein contaminants and UV 260 and 280 nm wavelength for DNA content determination.

### LOS immunochemical and physicochemical characterization

A panel of physicochemical and immunochemical methods for the complete characterization of gonococcal LOS was combined to fully characterize this antigen on different types of biological samples, such as bacterial lysates, outer membrane vesicles and extracted LOS samples.

### LOS quantification by semicarbazide derivatization and SE-HPLC analysis

The quantification of lipooligosaccharide content in GMMA samples was measured by SE-HPLC analysis by quantifying the reactive carbonyl groups of the saccharide moiety, generated after acid hydrolysis to remove the Lipid A and derivatized with semicarbazide, as previously reported^49,50^. The final quantified nmol of terminal KDO corresponds to the moles of LOS in *N. gonorrhoeae* biological samples. The KDO content of the GMMA samples is quantified based on a calibration curve prepared starting with a standard KDO ammonium salt solution. The LOS content is expressed in nmol/mL of KDO, which matches the nmol/mL of OS, and it is lastly reported as nmol_LOS_/mg protein, to normalize on the protein content.

### Silver stain

From a qualitative perspective, the glycoforms of LOS structures exposed by each strain were established by normalizing the quantity of heat-inactivated bacterial lysates (according to the OD_600nm_) and/or corresponding GMMA/extracted LOS samples (based on the LOS quantification titer by HPLC) and analyzing samples on 16% Tris–glycine gels using Tris–glycine 1x running buffer (Thermo Fisher Scientific). Then, after the fixation step using 5% acetic acid and 40% ethanol solution, an additional step of oxidation of the sugar with 0.7% sodium metaperiodate was added, finally staining using SilverQuest Staining kit (Thermo Fisher Scientific) according to the manufacturer’s recommendations. Silver-stained gel images were acquired using the Scanner EPSON PERFECTION V700 PHOTO.

This method detects oligosaccharide-derived relative masses, thus enabling the visualization of all the predominant LOS structures in the sample. The eight MS11 *lgt* mutant strains expressing defined LOS structures confirmed that different LOS structures could be differentiated using this method.

### Western Blot

Samples were further characterized by Western Blot, evaluating their reactivity with the combination of specific anti-LOS monoclonal antibodies. Heat-inactivated bacterial lysates, GMMA or extracted LOS were run on a 16% Tris–glycine SDS–PAGE gel using a Tris–glycine 1x buffer. LOS was transferred to nitrocellulose membranes (The iBlot Kit, Thermo Fisher Scientific), and membranes were blocked with PBS 1x + Tween20 0.05% + BSA 3% for 1 h at room temperature. Anti-LOS mAbs were incubated with membranes for 1 h at room temperature, and mAb-reactive LOS bands were visualized with anti-mouse IgG alkaline phosphatase secondary antibody, followed by reaction with the AP Conjugate Substrate kit (Biorad). Membrane images were acquired using the Scanner EPSON PERFECTION V700 PHOTO.

For the Western Blot analysis, mAb L3,7,9^51^, targeting the lacto-*N*-neotetraose structure, composed of four sugars extending from Heptose-I (Galβ1-4GlcNAcβ1-3Galβ1-4Glc, Fig. 2A, green box), and mAb 17-1-L1 (henceforth referred to as mAb L1), which binds to an alternative Heptose-I structure (Galα1-4Gal), thus recognizing the L1 meningococcal serotype, also known as the P^K^ structure (Galα1-4Galβ1-4Glc as α-chain, Fig. 2A, purple box), were selected. Furthermore, anti-gonococcal mAb 2C7 was implemented to detect the presence of lactose α-linked to Heptose-II (Fig. 2A, yellow box), with at least the first two sugars in the α-chain, but able to recognize structures with extensions from Heptose I beyond lactose, together with mAb 4C4, targeting highly truncated structures of LOS (Glcβ1-4Hep), were selected to finalize this panel, as reported in Fig. 2A, blue box. The anti-LOS mAb L1^24^, 4C4^25^, L3,7,9^24^ and 2C7^26^ have been described previously. Mouse mAb 2C7 has been produced internally as a recombinant mAb. Purified mAb 4C4 and supernatant of mAb L3,7,9 are commercially available (Novus Biologicals). Using these mAbs, which specifically react with different terminal sugar components of LOS, in Western Blot allowed us to precisely identify the prevalent LOS structure expressed in each strain.

### Immunization study

Animal husbandry and experiments were ethically reviewed and carried out in accordance with European Directive 2010/63/EU, in compliance with relevant guidelines (Italian Legislative Decree No. 26/2014) and GSK’s policy and guidelines on the care, welfare and treatment of animals, in GSK animal facilities located in Siena, Italy (AAALAC accredited). The ethical protocol P004/26/01 was reviewed by the local GSK ethical committee. The studies refer to research projects approved by the Italian Ministry of Health.

Female CD1 mice, 7-week-old, were purchased from Charles River Laboratories and kept in a controlled environment (individually ventilated cages; 22 ± 3 °C; 12 h/12 h light/dark cycle). Animals and their housing and husbandry were checked daily, and their well-being and health status were recorded in a dedicated logbook according to the local standard operating procedures. Final bleeding was performed under general anesthesia, and animals were euthanized by cervical dislocation before recovery from anesthesia.

CD1 mice (10/group) were immunized intraperitoneally twice on day 1 and day 29 with GMMA from different strains (F62 and SK92-679 strains) at a 10 μg protein-based dose in 200 μL adsorbed to Alum hydroxide (3 mg/mL). Mouse sera collected two weeks after the second dose (day 43) were analyzed in pools in functional assays as described below. In the in vivo immunization study using GMMA from MS11 mutant strains, female 7-week-old CD1 mice (10/group) were immunized intraperitoneally twice on day 1 and day 29. Different from the other study, GMMA were normalized based on LOS content at a dose of 1.5 nmol_LOS_ in 200 μL, adsorbed to Alum hydroxide (3 mg/mL). Mouse sera were collected two weeks after the second dose (day 43) and analyzed in pools in functional assays as described below.

### Human serum bactericidal assay (hSBA)

Bactericidal antibodies were measured by human serum bactericidal activity (hSBA) assay against the GC strains FA1090, F62, SK92-679, MS11, BG27, WHO-M, WHO-G and WHO-N using normal human serum from healthy donors as a complement source. Bacterial colonies from an overnight culture were resuspended in GC + 1% Isovitalex. Cytidine-5’-*O*-monophospho-*N*-acetylneuraminic acid [CMP-NANA] was added to the broth medium for serum-sensitive strains to render them resistant to killing by normal human serum: 0.5 µg/ml for F62 and 0.2 µg/ml for WHO-M and MS11 strains, and incubated at 37 °C with gentle shaking until the culture reached OD_600_ = 0.5. The broth culture was then diluted 1:10,000 in SBA buffer (Dulbecco’s phosphate-buffered saline [dPBS] + 1% BSA + 0.1% glucose) with the exception of the BG27 strain, which was diluted 1:2500. Mouse sera, previously heat-inactivated at 56 °C for 30 min, were serially diluted in SBA buffer. The assay was assembled in a sterile 96-flat-bottom well microplate in a final volume of 32 µL/well. The serial dilutions of each test sample were allowed to react with pre-diluted bacteria and with human serum from healthy donors as a complement source (16% for FA1090, SK92-679, BG27, WHO-G, WHO-N and 10% for F62, MS11, WHO-M). The reaction mixture was incubated at 37 °C for 60 min at 160 rpm, then agar overlay medium was added and the plate was incubated overnight at 37 °C with 5% CO_2_ in a humid atmosphere. One day later, colony-forming units (CFUs) were manually counted or automatically acquired with a high-throughput image analysis system (Discovery v12 Axiolab or ScanLab) and were automatically counted for each well by an image analysis system (Reading AxioVision or the internally developed software). Bactericidal titer was defined as the reciprocal of the serum dilution giving a killing >50% with respect to the average number of CFU calculated in replicates of ‘without serum’ control. The bactericidal titer for each test sample was calculated as the reciprocal of the serum dilution giving a killing >50% respect to the average number of CFU calculated on the 8 replicates without serum control at *T*_60min_ (average CFU without serum control). Where more than one serum dilution gives 50% < killing < 55%, the lowest dilution is chosen to calculate the hSBA titer. The Mann–Whitney *U*-test was used to statistically compare hSBA titers obtained for experimental replicates from different immunization groups.

### Competitive human serum bactericidal assay

The competitive human serum bactericidal assay (hSBA) was used to measure the residual serum bactericidal activity of a pool of immunized mouse sera, known to give high hSBA titers, after incubation with different competitors. These experiments allowed us to understand the role of anti-LOS antibodies in the SBA response elicited by a GMMA-based vaccine. Due to the limited availability of sera reagents at adequate volumes, the competitive SBA experiments were performed only once. A fixed dilution of the tested pool (sera obtained from mice immunized with GMMA vaccine) was incubated 1:1 (vol/vol) with 3 different concentrations (in terms of nmol_LOS_/mL) of each competitor for 1 h at 37 °C, 180 rpm. The same pool was also incubated 1:1 (vol/vol) with SBA buffer to measure the hSBA titer of “*not inhibited*” sample. After 1 h of incubation, the mixture serum-competitor was dispensed in a plate, diluted 1:2 for 11 dilution-steps and then bacteria and human complement were added following the hSBA assay protocol. The “*no serum*” control (8 wells) was included in each plate, and it comprises bacteria mixed with active human complement (AC) in the absence of a serum sample. This control is used to exclude complement toxicity and to determine 100% bacterial growth.

### Bacterial Adhesion Inhibition (BAI) assay

A cell-based fluorescent BAI assay was used to assess the capacity of the tested murine pooled sera to inhibit the adhesion of FA1090 and SK92-679 strains to SV-HUC1 human urethral epithelial cells.

Cultured cells were detached from a 175 cm^2^ flask after resuspension to avoid cell clumping. Cell number and viability were determined by an automated counter. They were then seeded into 96-well plates (3 × 10^4^ cells/well) and cultured in F-12K Nut Mix medium till confluence. GC strains were harvested from a fresh overnight plate culture into 10 ml GC + 1% Isovitalex medium. Bacteria were grown at 37 °C under shaking till OD_600_ = 0.5, then resuspended in DPBS and labeled with Oregon Green dye for 15 min at 37 °C. Afterwards, bacteria were washed to remove excess dye, resuspended in DPBS–2% BSA, and combined, at final OD_600_ = 0.1, with an equal volume of serially cell medium-diluted sera for 15 min at RT. Bacteria-sera complexes were added to cell plates and incubated for 1 h at 37 °C to allow bacterium-cell adhesion. After three washings with DPBS, samples were fixed for 20 min with 4% formaldehyde at room temperature and, after one washing step with DPBS, finally one volume of distilled water was added to each well. Plates were analyzed by the Opera Phenix instrument (Revvity). Due to variability in the results obtained with the negative control (Alum), a threshold of 30% was set to discriminate between positive samples and Alum itself. This 30% cutoff was defined based on over 100 observations during the setup of the BAI assay, where Alum serum showed bacterial inhibition lower than 15% in around 95% of the observations and lower than 30% in 100% of the observations.

## Supplementary information

Supplementary Information

## Data Availability

All data generated or analyzed during this study are included in this published article. There are restrictions on the availability of NgG and related items in this work because of multiple company-owned patent related to these products.
