# Supplementary material for: Contribution of the different Neisseria gonorrhoeae lipooligosaccharide structural variants to functional responses elicited by GMMA outer membrane vesicles
Source: NPJ Vaccines. 2025 Oct 30;10:223. doi: 10.1038/s41541-025-01271-1 (PMC12575615; doi:10.1038/s41541-025-01271-1)
Supplement: Supplementary file 1 — Supplementary Information [file 41541_2025_1271_MOESM1_ESM.pdf]

Supplementary Information

Tables

Suppl. Table 1. Dose of each MS11 Δ*lpxL1 lgt* mutant GMMA in terms of proteins.

GMMA MS11 Δ*lpxL1*	LOS/Protein ($nmol_{KDO}/mg_{protein}$)	Dose ($nmol_{LOS}$)	Dose ($\mu g_{protein}$)
**2HexG+**	194.8	1.5	7.7
**2HexG-**	250.4	1.5	5.9
**3HexG+**	247.0	1.5	6.1
**3HexG-**	226.0	1.5	6.6
**4HexG+**	238.4	1.5	6.3
**4HexG-**	386.9	1.5	3.9
**5HexG+**	208.1	1.5	7.2
**5HexG-**	156.4	1.5	9.6

Suppl. Table 2. List of primers used for the generation of *lpxL1* knockout mutants in *N. gonorrhoeae* F62, SK92-679 and MS11 v.4/3/1 LOS mutant strains.

**Name**	**Sequence 5'-3'**
LpxL1 UP Fwd	GCATTTGTATTTTGCCGTCTG
LpxL1 DO Rev	CGCCATTTTCTACGCTTTGCCAAG
NGO_lpxL1_wtcheck-Rev	GCGGAACTGTTTGACGAG
LpxL1 est Fwd	CCGCCAAACTCAATCCTTCG
LpxL1 est Rev	GCAAACTTTTGTTTCACCGTTTCCG

Figures

Bacterial lysates of gonococcal isogenic MS11 mutant strains

		2HexG+	2HexG-	3HexG+	3HexG-	4HexG+	4HexG-	5HexG+	5HexG-
Silver-stained SDS-PAGE	3.5 KDa	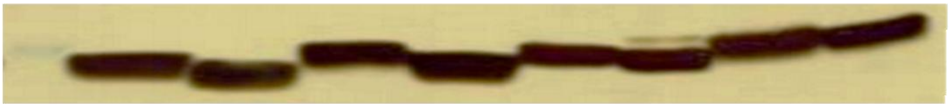							
Western Blot with anti-LOS mAbs	mAb 2C7	+	-	+	-	+	-	+	-
	mAb L3,7,9	-	-	-	-	-	+	-	-
	mAb 4C4	-	+	-	-	-	-	-	-
	mAb L1	-	-	+	+	-	-	-	-

Suppl. Figure 1 LOS immunochemical characterization of GMMA from the eight isogenic MS11 *Neisseria gonorrhoeae* strains. It is important to notice that, as reported by Chakraborti *et al*. [1] merely fixing an enzyme does not guarantee that all LOS on the bacterial surface will be completely substituted with the glycan added by the encoded Lgt enzymes. Therefore, the same mutant strain can express the expected LOS structure together with minimal impurities of shorter structure depending on the amount and efficiency of each Lgt enzyme.

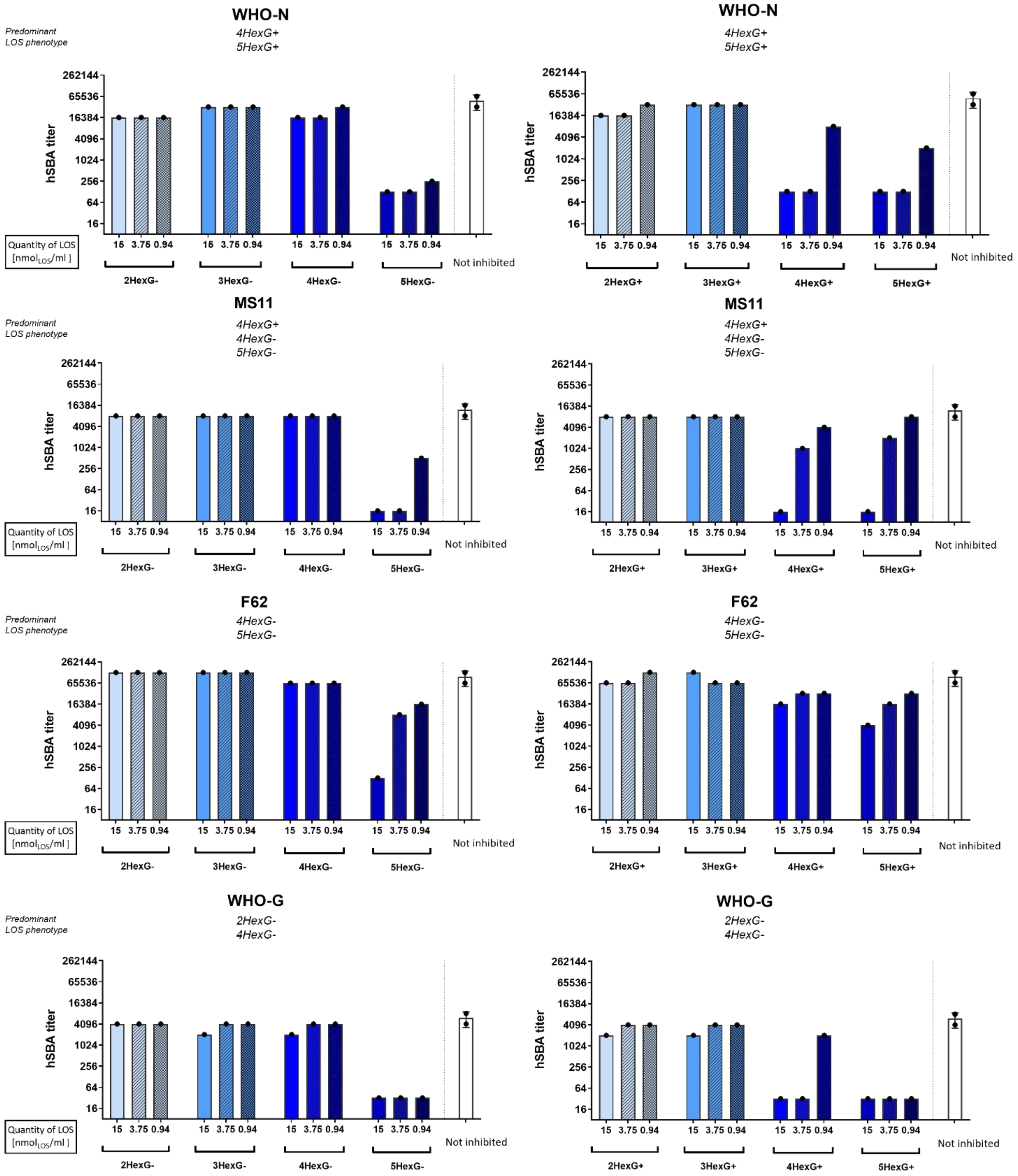

Suppl. Figure 2 Competitive hSBA titers obtained using sera from mice immunized with GMMA FA1090 Δ*lpxL1* Δ*rmp* (NgG) after preincubation with 3 different concentrations in terms of LOS content (15, 3.75 and 0.94 $nmol_{LOS}$/ml) of GMMA from MS11 mutant strains. Each bar/dot represents the titer obtained using a pool of sera from 10 mice immunized with GMMA FA1090 Δ*lpxL1* Δ*rmp* and competed with GMMA from the MS11 mutants reported in the x-axis. The white bar represents the uninhibited sample, tested in duplicate.

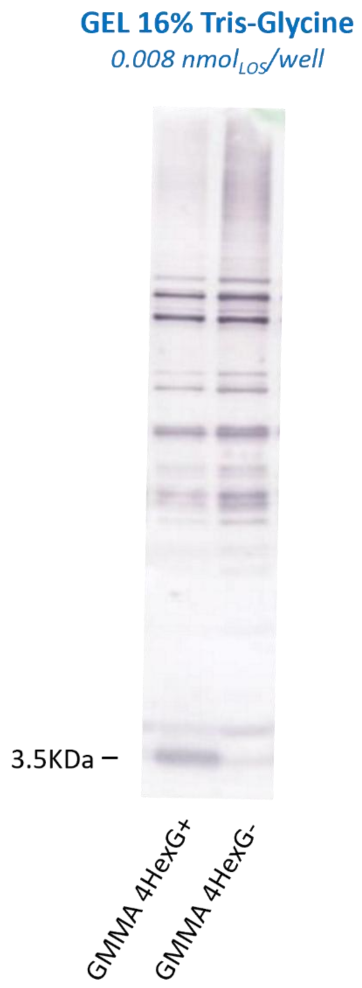

Suppl. Figure 3 Western Blot analysis of IgG antibodies elicited by immunization with the GMMA FA1090 vaccine candidate against GMMA MS11 4HexG+ and GMMA MS11 4HexG-.

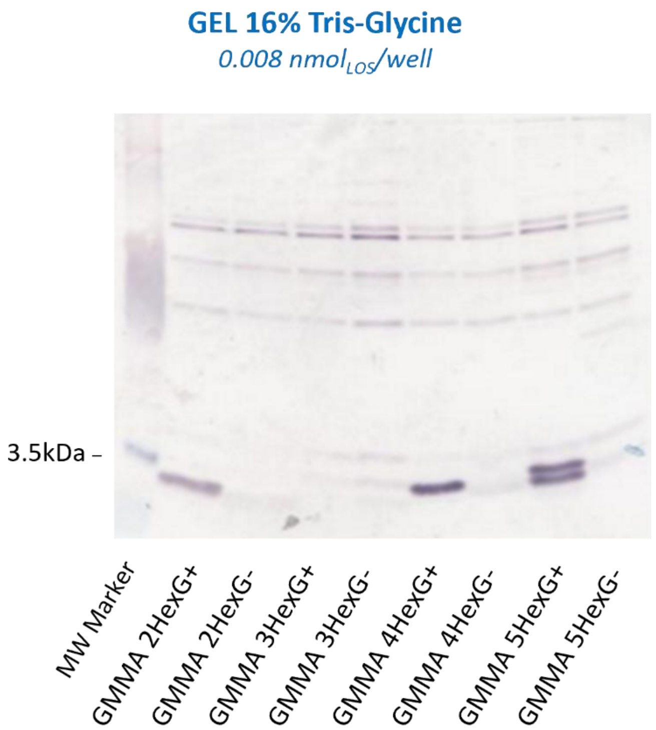

Suppl. Figure 4 Western Blot analyses of IgG antibodies elicited by immunization with GMMA MS11 Δ*lpxLI* 4HexG+ on the different GMMA MS11 mutants.

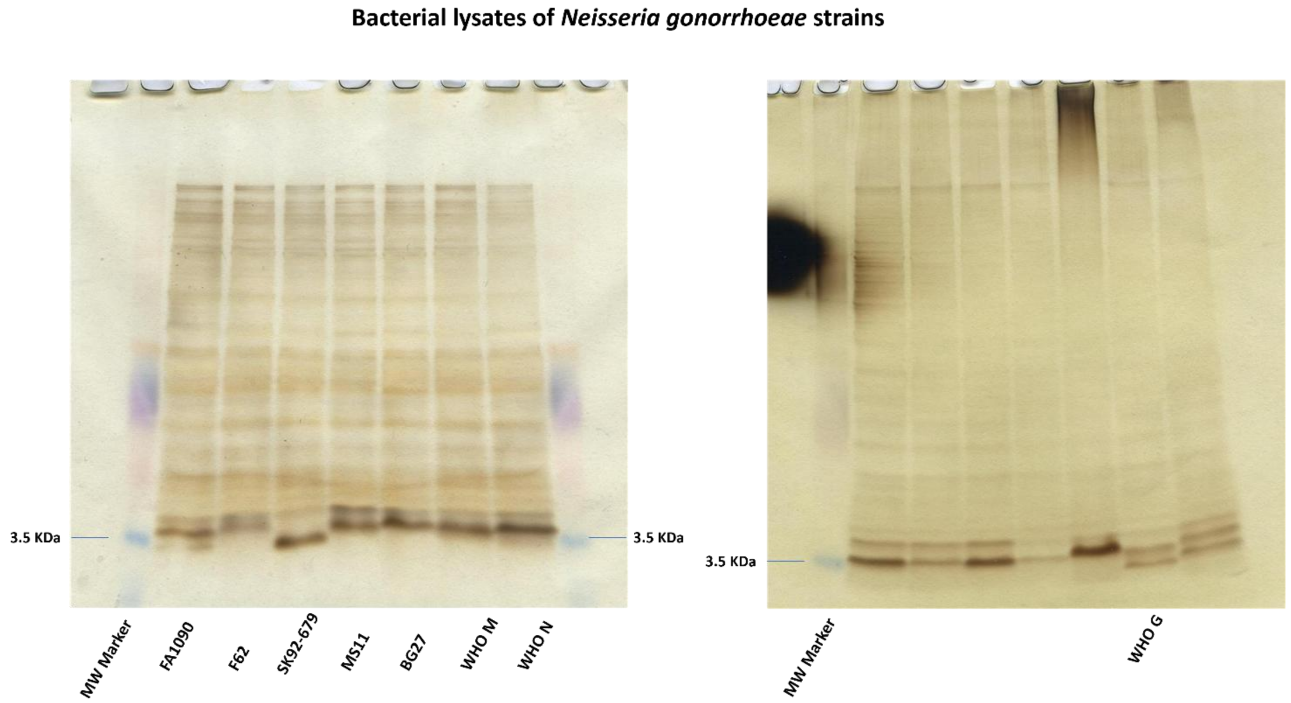

Suppl. Figure 5 Uncropped and unprocessed scans of the silver-stained SDS-PAGE used to generate Figure 2.

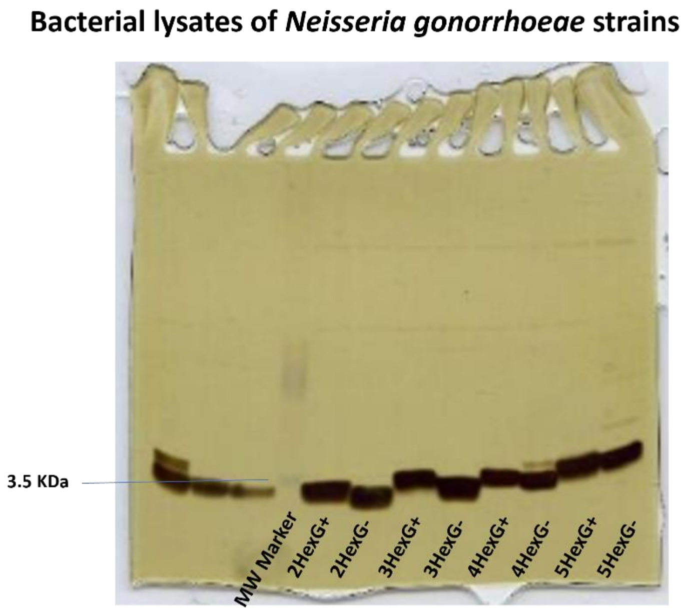

Suppl. Figure 6 Uncropped and unprocessed scan of the silver-stained SDS-PAGE used to generate Supplementary Figure 1.

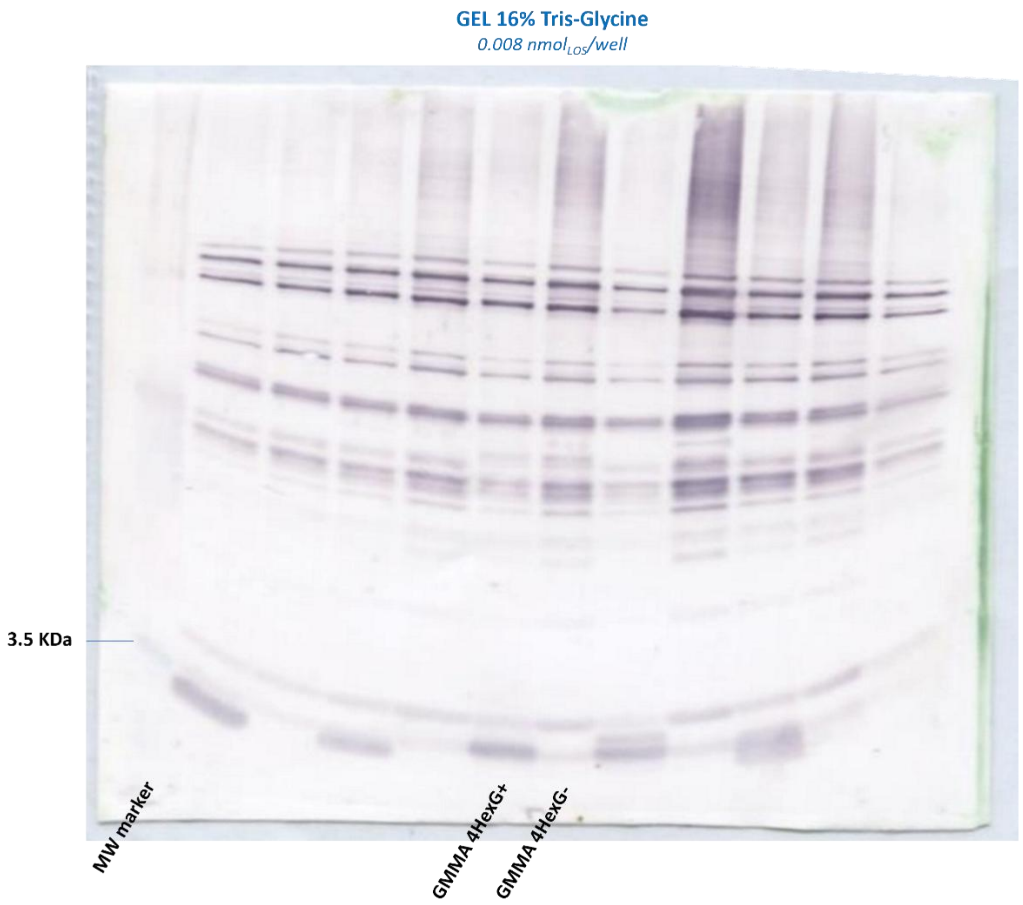

Suppl. Figure 7 Uncropped and unprocessed scan of the Western blot used to generate Supplementary Figure 3.

Reference

1. Chakraborti, S., et al., *Phase-Variable Heptose I Glycan Extensions Modulate Efficacy of 2C7 Vaccine Antibody Directed against Neisseria gonorrhoeae Lipooligosaccharide*. J Immunol, 2016. **196**(11): p. 4576-86.
